# Redefining the Diagnostic and Therapeutic Landscape of Non-Small Cell Lung Cancer in the Era of Precision Medicine

**DOI:** 10.3390/jcm14228021

**Published:** 2025-11-12

**Authors:** Shumayila Khan, Saurabh Upadhyay, Sana Kauser, Gulam Mustafa Hasan, Wenying Lu, Maddison Waters, Md Imtaiyaz Hassan, Sukhwinder Singh Sohal

**Affiliations:** 1 International Health Division, Indian Council of Medical Research, Ansari Nagar, New Delhi 110029, India; 2 St. Jude Children’s Research Hospital, Memphis, TN 38105, USA; 3 Department of Biosciences, Jamia Millia Islamia, New Delhi 110025, India; sana1900562@st.jmi.ac.in; 4 Department of Biochemistry, College of Medicine, Prince Sattam Bin Abdulaziz University, Al-Kharj 11942, Saudi Arabia; 5 Respiratory Translational Research Group, Department of Laboratory Medicine, School of Health Sciences, College of Health and Medicine, University of Tasmania, Launceston, TAS 7249, Australia; 6 St Vincent’s Hospital, Melbourne, VIC 3065, Australia; 7 Centre for Interdisciplinary Research in Basic Sciences, Jamia Millia Islamia, New Delhi 110025, India

**Keywords:** non-small cell lung cancer, cancer diagnostics, targeted therapy, immunotherapy, AI in oncology, biomarker discovery, cancer vaccines, precision medicine

## Abstract

Non-small cell lung cancer (NSCLC) remains a leading cause of cancer-related mortality globally, driven by marked molecular and cellular heterogeneity that complicates diagnosis and treatment. Despite advances in targeted therapies and immunotherapies, treatment resistance frequently emerges, and clinical benefits remain limited to specific molecular subtypes. To improve early detection and dynamic monitoring, novel diagnostic strategies—including liquid biopsy, low-dose computed tomography scans (CT) with radiomic analysis, and AI-integrated multi-modal platforms—are under active investigation. Non-invasive sampling of exhaled breath, saliva, and sputum, and high-throughput profiling of peripheral T-cell receptors and immune signatures offer promising, patient-friendly biomarker sources. In parallel, multi-omic technologies such as single-cell sequencing, spatial transcriptomics, and proteomics are providing granular insights into tumor evolution and immune interactions. The integration of these data with real-world clinical evidence and machine learning is refining predictive models and enabling more adaptive treatment strategies. Emerging therapeutic modalities—including antibody–drug conjugates, bispecific antibodies, and cancer vaccines—further expand the therapeutic landscape. This review synthesizes recent advances in NSCLC diagnostics and treatment, outlines key challenges, and highlights future directions to improve long-term outcomes. These advancements collectively improve personalized and effective management of NSCLC, offering hope for better-quality survival. Continued research and integration of cutting-edge technologies will be crucial to overcoming current challenges and achieving long-term clinical success.

## 1. Introduction

Lung cancer remains a formidable public health challenge worldwide, accounting for the highest number of cancer-related deaths. The global burden of lung cancer is well documented, with millions of new cases diagnosed annually. Within this group, non-small cell lung cancer (NSCLC) comprises approximately 85% of all lung cancer cases [1], underscoring its clinical and scientific significance [2]. The substantial mortality and morbidity associated with NSCLC necessitate a thorough understanding of its pathology, diagnostic modalities, and treatment options.

Over the past few decades, the landscape of NSCLC management has undergone a remarkable transformation. Historically, systemic chemotherapy was the mainstay for advanced-stage lung cancer, providing modest but measurable survival benefits. At the same time, palliative radiation was used primarily for symptom relief without a significant impact on overall survival. The advent of precision oncology, however, has transformed treatment approaches, enabling targeted therapies and immunotherapies that can significantly improve outcomes in molecularly defined patient subsets. The integration of molecular diagnostics, including next-generation sequencing, has enabled the identification of key oncogenic drivers such as Epidermal Growth Factor Receptor (*EGFR*) mutations, Anaplastic Lymphoma Kinase (*ALK*) rearrangements, and Kirsten Rat Sarcoma Viral Oncogene Homolog (*KRAS*) mutations, among others [3]. These advances have paved the way for the development and clinical implementation of targeted therapies that have significantly improved patient outcomes.

Moreover, the advent of immunotherapy has further redefined treatment paradigms in NSCLC. Immune checkpoint inhibitors targeting Programmed Cell Death Protein-1 or its ligand (*PD-1* or *PD-L1*) have demonstrated notable efficacy in subsets of patients, providing durable responses and altering the natural history of the disease [4,5,6]. The combination of targeted agents with immunotherapeutic approaches, along with emerging surgical strategies such as local consolidative pulmonary resection in stage IV NSCLC with oncogenic drivers, and advances in radiotherapy, contributes to the evolving multimodal management of NSCLC [7].

The objective of this review is to comprehensively examine the latest advances in the pathology and therapeutics of NSCLC. We aim to provide an updated synthesis of the current literature, with a focus on recent developments in early diagnostics, histopathological refinements, and novel treatment strategies. By integrating traditional clinicopathological evaluations with cutting-edge molecular and immunological insights, this review seeks to bridge the gap between basic research and clinical application. In doing so, we highlight the translational potential of emerging techniques and underscore the importance of multidisciplinary collaboration in advancing patient care.

In this review, we focus on connecting diagnostic approaches with subsequent therapeutic decisions in NSCLC, emphasizing how new methods inform treatment planning rather than listing isolated advances. We discuss how tools like liquid biopsy and imaging biomarkers support patient selection for perioperative and adjuvant therapies, how multi-omic and spatial profiling reveal mechanisms behind therapy resistance, and how insights from clinical practice help translate trial findings into real-world management. By integrating diagnostics with treatment strategy, this review aims to provide a practical framework for guiding precision care and optimizing treatment sequencing.

## 2. Non-Small Cell Lung Cancer

NSCLC represents a heterogeneous group of epithelial lung malignancies that differ significantly from small cell lung cancer (SCLC) in terms of histopathological features, molecular characteristics, biological behavior, and therapeutic responsiveness. While SCLC is characterized by its neuroendocrine origin, rapid proliferation, and early metastatic potential, NSCLC typically follows a relatively indolent course compared with SCLC, with greater heterogeneity in both morphology and molecular drivers, allowing for more targeted therapeutic interventions [8,9].

NSCLC comprises approximately 85% of all lung cancers and is histologically classified into three major subtypes: adenocarcinoma, squamous cell carcinoma, and large cell carcinoma. Among these, adenocarcinoma is the most prevalent subtype globally, especially among non-smokers and younger patients, and typically arises in the peripheral regions of the lung. Histologically, it displays glandular differentiation and mucin production. Subtypes of adenocarcinoma, including acinar, lepidic, papillary, micropapillary, and solid patterns, are defined based on their architectural morphology and have prognostic significance [10]. Immunohistochemically, adenocarcinomas are usually positive for markers such as thyroid transcription factor-1 (TTF-1) and Napsin A [11].

Squamous cell carcinoma, the second most common subtype, has a strong association with tobacco smoking and generally originates in the central bronchi. Histological features include keratinization and intercellular bridges, and immunohistochemical markers such as p40, p63, and cytokeratin 5/6 aid in its diagnosis [12]. Squamous cell carcinomas are more likely to present with cavitation and are frequently associated with obstructive symptoms due to their central location [13].

Large cell carcinoma, though less frequent, is a poorly differentiated neoplasm. It is typically a diagnosis of exclusion, made based on the absence of squamous, glandular, or small cell differentiation. This subtype often demonstrates aggressive clinical behavior and is commonly diagnosed at advanced stages [14]. From a molecular perspective, NSCLC exhibits a wide array of genetic and epigenetic alterations. Adenocarcinomas frequently harbor mutations in *EGFR*, *KRAS*, B-Raf proto-oncogene (*BRAF*), and rearrangements involving *ALK*, c-ros oncogene 1 (*ROS1*), and rearranged during transfection (*RET*), which serve as critical predictive biomarkers and therapeutic targets [15,16]. In contrast, squamous cell carcinomas demonstrate a higher frequency of *TP53* mutations, *CDKN2A* deletions, and *PIK3CA* alterations, although they currently have fewer actionable molecular targets [17].

The distinct cellular origins, molecular alterations, and clinical behaviors of NSCLC subtypes not only shape their pathological classification but also carry direct implications for treatment planning. As the therapeutic landscape shifts toward precision oncology, this molecular heterogeneity underscores the importance of thorough diagnostic workups, including histological and genetic profiling, to tailor interventions effectively. Recent molecular profiling efforts and pan-cancer analyses have revealed previously unrecognized NSCLC subsets driven by rare gene fusions, copy number variations, and epigenetic dysregulation, expanding the spectrum of potentially actionable alterations. These insights are now being integrated into adaptive trial platforms and biomarker-driven screening programs, marking a shift from purely morphological to multi-omic disease classification.

However, beyond tumor biology, a comprehensive understanding of the disease also requires close attention to the environmental, genetic, and immunological factors that predispose individuals to NSCLC. Exploring these etiological determinants is essential for identifying high-risk populations and informing strategies for prevention and early detection.

## 3. Risk Factors and Etiological Insights

The etiology of NSCLC is multifactorial, involving a complex interplay between environmental exposures, genetic susceptibility, and host immune responses. While tobacco smoke remains the predominant carcinogen linked to lung cancer, emerging data has highlighted several non-smoking-related pathways that contribute to disease pathogenesis, especially in never-smokers [18]. Understanding these factors is crucial for identifying at-risk populations and developing preventative and early diagnostic strategies.

### 3.1. Environmental and Lifestyle Risk Factors

Cigarette smoking continues to be the most significant environmental risk factor for NSCLC, accounting for approximately 85–90% of all lung cancer cases globally [19]. Tobacco smoke contains numerous carcinogens, including nitrosamines and polycyclic aromatic hydrocarbons, which cause DNA adduct formation and mutational signatures characteristic of smoking-related NSCLC, particularly in squamous cell carcinoma and small cell histologies [10]. Despite a global decline in smoking prevalence, there has been a parallel rise in the use of alternative nicotine delivery systems such as e-cigarettes and heated tobacco products. Although their long-term impact is not yet fully understood, early evidence suggests that these products may still induce inflammatory and mutagenic effects in airway epithelium, thereby sustaining carcinogenic risk [20].

Occupational and environmental exposures also contribute to lung carcinogenesis. Inhalation of asbestos fibers is a well-established risk factor, particularly in individuals with prolonged occupational exposure, where the risk is further amplified by concurrent smoking [21]. Exposure to diesel exhaust particles and silica dust, common in industrial and construction settings, has been linked to increased lung cancer incidence, primarily through mechanisms of oxidative stress and chronic inflammation [22]. Environmental factors such as radon—a naturally occurring radioactive gas—and particulate matter (PM2.5) from urban air pollution have been associated with lung cancer in both smokers and never-smokers, implicating ambient exposures in global lung cancer burden [23,24,25].

### 3.2. Genetic and Molecular Risk Factors

Although most NSCLC cases are sporadic, a small proportion exhibit familial clustering, suggesting an inherited genetic susceptibility. Germline mutations in genes involved in DNA repair and cell cycle regulation have been identified in select families, although these remain relatively rare [26]. On the other hand, somatic mutations in genes such as *EGFR*, *KRAS*, *ALK*, *ROS1*, *RET*, and *MET* serve as oncogenic drivers in a significant subset of NSCLC. They are critical determinants of both tumor biology and therapeutic response [27]. Interestingly, the distribution of these mutations varies by ethnicity. For example, *EGFR* mutations are found in nearly 50% of Asian non-smoking adenocarcinoma patients, compared to 10–15% in Western populations, suggesting a gene–environment interaction that modulates risk [28].

### 3.3. Immunological and Microenvironmental Contributors

Beyond mutational events, the tumor microenvironment plays a crucial role in the initiation and progression of NSCLC. Chronic pulmonary inflammation—resulting from infections, autoimmune conditions, or environmental insults—creates a permissive environment for DNA damage and malignant transformation [29]. Furthermore, NSCLC tumors often exhibit mechanisms of immune evasion, such as the upregulation of immune checkpoint molecules (PD-L1), the recruitment of regulatory T cells, and the exclusion of cytotoxic T cells, which enable unchecked proliferation and resistance to immune surveillance [30]. PKM2 isoenzyme controls pyruvate flux and nuclear signaling pathways in the Warburg effect. In this mechanism, cancer cells use glycolysis and lactic acid fermentation to produce energy, implicating it as a critical driver of metabolic adaptation in NSCLC [31,32]. These immunological dynamics not only contribute to tumorigenesis but also shape the response to immunotherapeutic interventions.

## 4. Stage Classification of NSCLC

The staging of NSCLC plays a pivotal role in determining prognosis, guiding therapeutic decisions and evaluating treatment outcomes. The most widely used system for staging NSCLC is the Tumor, Node, Metastasis (TNM) classification, currently in its 8th edition as established by the Union for International Cancer Control (UICC) and the American Joint Committee on Cancer (AJCC) [33].

The forthcoming 9th edition of the TNM classification introduces key refinements over the 8th edition, particularly in nodal staging. The subdivision of N2 disease into N2a (single-station) and N2b (multi-station) reduces prognostic heterogeneity and provides more accurate survival prediction, as demonstrated by Demirdöğen et al. [34]. Their findings show that N2b disease independently increases 2-year mortality risk compared to N2a, highlighting the clinical relevance of detailed nodal assessment using EBUS-TBNA. Similarly, Wu et al. [35] validated the reclassification of select T1N2 cases from stage IIIA to IIB (N2a2 subgroup), suggesting that this revision more accurately reflects survival outcomes and may refine decisions regarding adjuvant therapy and trial eligibility. Together, these updates enhance diagnostic precision and may guide stage-adapted multimodal strategies in NSCLC.

Imaging plays a central role in staging. Contrast-enhanced computed tomography (CT) is routinely employed for initial assessment of the thorax and upper abdomen. Positron emission tomography (PET) combined with CT (PET-CT) is instrumental in detecting metabolically active tumours, lymph nodes, and distant metastases, particularly in the brain, bones, and adrenal glands [36]. Magnetic resonance imaging (MRI), especially brain MRI, is often indicated in cases with neurological symptoms or when PET findings are equivocal. These imaging modalities, in conjunction with endobronchial ultrasound-guided (EBUS) nodal sampling and mediastinoscopy, help establish a more accurate and comprehensive staging profile [37].

The prognostic implications of staging are profound. Patients with stage I disease may benefit from curative-intent surgical resection, while those with stage II–III often require multimodal treatment including chemotherapy, radiation, or immunotherapy. Stage IV disease, indicating metastatic spread, typically necessitates systemic therapy with a focus on targeted agents or immune checkpoint inhibitors based on molecular profiling. Thus, accurate staging remains a cornerstone of personalized NSCLC management and directly impacts clinical outcomes [38].

## 5. Emerging Paradigms in Histopathological Characteristics and Diagnostic Refinement

The histopathological assessment of NSCLC has evolved significantly beyond traditional morphology-based classification, incorporating immunohistochemistry, molecular diagnostics, and, more recently, computational and AI-assisted tools. This integrated approach is essential, not only for accurate tumor subtyping, but also for identifying therapeutically actionable alterations, understanding resistance mechanisms, and optimizing personalized treatment strategies.

### 5.1. Classical Histological Subtypes

NSCLC comprises diverse histological entities with distinct morphological and immunohistochemical profiles. Adenocarcinoma is the most prevalent subtype, particularly among never-smokers, and typically displays glandular differentiation and mucin production. Histological patterns include acinar, papillary, lepidic, micropapillary, and solid forms, each with distinct prognostic implications. For instance, the lepidic pattern is associated with indolent behavior, whereas micropapillary and solid patterns correlate with aggressive disease and poorer outcomes [39]. Immunohistochemical staining for TTF-1 and Napsin A is commonly employed to confirm adenocarcinoma lineage, especially in poorly differentiated tumors where morphology alone is insufficient [40].

In contrast, squamous cell carcinoma is characterized histologically by keratinization, intercellular bridges, and the presence of squamous pearls. These tumors are centrally located and strongly associated with smoking history. Immunohistochemically, they are typically positive for p40, CK5/6, and p63, which assist in distinguishing them from adenocarcinomas, particularly in small biopsies where tumor architecture may be unclear [41].

Large cell carcinoma is a diagnosis of exclusion, defined by the absence of glandular or squamous differentiation on both morphological and immunohistochemical examination. These tumors often appear as poorly differentiated, undifferentiated neoplasms and pose significant diagnostic challenges, especially in small biopsies. Variants such as large cell neuroendocrine carcinoma or basaloid carcinoma add further complexity, requiring careful histological and immunophenotypic evaluation [42].

### 5.2. Role of Advanced Pathology in Modern Medicine

With the increasing complexity of NSCLC classification and the need for multiplex biomarker evaluation, pathology has embraced several advanced technologies. Multiplex immunohistochemistry (IHC) and immunofluorescence platforms enable the simultaneous visualization of multiple markers within a single tissue section, preserving spatial context and facilitating the differentiation of tumor heterogeneity and immune microenvironment features [43]. This is particularly useful in characterizing tumors with mixed features or unusual presentations.

Digital pathology, enhanced by AI algorithms, is gaining traction as a tool for both research and clinical applications. AI-driven systems can perform high-throughput analysis of histological slides, quantify tumor-infiltrating lymphocytes, and even predict molecular alterations from routine H&E (hematoxylin and eosin) images with increasing accuracy [44]. These tools offer the potential to improve diagnostic consistency, reduce inter-observer variability, and provide real-time decision support.

### 5.3. Next-Generation Sequencing in Histological Context

The integration of next-generation sequencing (NGS) with histopathological assessment represents a major advancement in the diagnostic workup of NSCLC. Molecular profiling is now considered essential for all advanced-stage non-squamous NSCLC cases and increasingly in squamous tumors, particularly among never-smokers or younger patients. Comprehensive NGS panels can detect a wide spectrum of alterations, including single-nucleotide variants, gene fusions, insertions/deletions, and copy number alterations across key driver genes such as *EGFR*, *ALK*, *ROS1*, *RET*, *BRAF*, and *MET* [45].

Furthermore, NGS plays a crucial role in identifying resistance mechanisms, such as T790M in *EGFR*-mutated tumors or *MET* amplification following *ALK* inhibitor therapy, thereby facilitating appropriate sequencing of targeted therapies [46]. In this context, the histogenomic approach enables a more nuanced understanding of tumor biology by correlating morphological features with underlying molecular drivers. The integration of classical histopathology with advanced diagnostic tools is central to the current paradigm of NSCLC management, allowing not only precise classification but also informed therapeutic choices tailored to the molecular landscape of each tumor.

## 6. Evolving Landscape of Early Diagnosis

Early detection remains the cornerstone of improving outcomes in NSCLC, where prognosis is closely linked to stage at diagnosis. However, traditional methods, such as imaging and tissue biopsy, have limitations in terms of sensitivity, accessibility, and applicability in the early stages of disease. In recent years, technological advances have led to the emergence of non-invasive diagnostic approaches, including liquid biopsies, radiomics-integrated low-dose CT (LDCT), and multi-analyte biomarker panels. These tools are reshaping the landscape of early detection by enabling real-time, minimally invasive cancer surveillance. This section critically examines recent breakthroughs in these modalities, emphasizing their clinical relevance and translational challenges.

### 6.1. Liquid Biopsy Approaches

Non-invasive “liquid biopsies” have emerged as a powerful tool for detecting tumor-specific genomic alterations in peripheral blood. In the context of lung cancer, circulating tumor DNA (ctDNA)-based assays enable the identification of clinically targetable mutations, such as *EGFR*, *ALK*, and *KRAS*, without the need for a tissue biopsy [47]. ctDNA serves two conceptually distinct clinical functions in NSCLC and should be described separately. In advanced disease, plasma ctDNA is primarily used for driver detection and real-time genotyping to guide targeted therapy selection and to identify emergent resistance mutations; assay sensitivity for this use is generally adequate because tumor shedding is higher in metastatic disease, and commercially validated NGS ctDNA platforms demonstrate acceptable concordance with tissue genotyping for common drivers in advanced NSCLC [48].

By contrast, ctDNA for minimal residual disease (MRD) detection after curative-intent therapy is a different use case that faces intrinsic sensitivity limits. Post-operative MRD levels are often very low (variant allele fractions frequently < 0.1%), causing suboptimal sensitivity in many assays despite high specificity; landmark and surveillance strategies both show promising prognostic value but variable sensitivity across studies [49]. Before ctDNA-based MRD assessment can be reliably used to escalate or de-escalate adjuvant therapy, further validation is essential. Large prospective MRD-guided clinical trials are needed to determine how changes in ctDNA levels should influence treatment decisions and patient outcomes. In addition, there must be standardization of assay methodologies—including the platforms used, limits of detection, timing and frequency of blood sampling, and reporting criteria—to ensure that MRD results are consistent and comparable across laboratories. Without such harmonization, there is a risk of false reassurance from undetected low-level disease or unnecessary treatment triggered by assay variability rather than true residual disease. While circulating tumor DNA–based assays are increasingly being explored for MRD detection and monitoring, most applications in NSCLC remain investigational. They are yet to be validated for routine clinical use.

Moreover, the integration of ctDNA into trial design allows for dynamic patient stratification, enrichment of high-risk cohorts, and the potential use of ctDNA clearance as an early surrogate endpoint, thereby reducing trial size and duration while enhancing statistical power [50]. Commercially available liquid-biopsy platforms such as *Guardant360 CDx* (circulating tumor DNA–based assay; FDA-approved as a companion diagnostic) and *FoundationOne Liquid CDx* (comprehensive genomic profiling assay; FDA-approved) exemplify clinically validated tools in this domain, while several others are currently being investigated.

However, their clinical utility for detecting MRD in nonmetastatic settings remains under investigation. Technological advancements have improved sensitivity, yet ctDNA detection in patients with low tumor burden continues to pose challenges due to variability in tumor DNA shedding, limited sampling frequency, and the need for ultra-deep sequencing. Several commercial assays are now being evaluated in ongoing trials to optimize MRD detection and guide adjuvant therapy decisions [51].

In addition to ctDNA and circulating tumor cells (CTCs), extracellular vesicles (EVs), especially exosomes, have emerged as rich sources of tumor-derived molecular cargo, including microRNAs (miRNAs), messenger RNAs (mRNAs), long non-coding RNAs (lncRNAs), and proteins [16]. These vesicles protect their contents from degradation, making them stable and accessible biomarkers in various body fluids such as blood, urine, saliva, and pleural fluids. Exosomal miRNAs—such as miR-216b, miR-210-5p, miR-451a, miR-185-5p, miR-1290, and miR-146a-5p—have been shown to differentiate lung cancer patients from healthy individuals and are good diagnostic biomarker candidates [52].

Furthermore, recent advances in serological assays have enabled the detection of tumor-associated autoantibodies (TAAbs) as potential biomarkers for the early diagnosis of lung cancer. These autoantibodies, which target tumor-associated antigens (TAAs) such as p53, NY-ESO-1, SOX2, CAGE, annexin 1 and GBU4-5 have demonstrated promising diagnostic potential. A notable multi-cohort study reported sensitivities ranging from 36% to 39% and specificities between 89% and 91% across three independent lung cancer cohorts using such autoantibody panels, underscoring their utility in high-risk populations for prescreening purposes [53].

Recent efforts also explore methylation profiling of cell-free DNA (cfDNA), which can provide tumor-specific epigenetic fingerprints. Multi-cancer early detection (MCED) assays, such as *Galleri* (Grail Inc.), utilize targeted methylation analysis of cell-free DNA to detect cancer signals and accurately predict the tissue of origin across over 50 cancer types, including lung malignancies [54]. In a large independent validation substudy involving 4077 participants, the MCED test demonstrated a specificity of 99.5% and correctly identified the origin of cancer in 88.7% of true-positive cases. While sensitivity increased with advancing stage, the findings highlight its complementary potential to traditional single-cancer screening tools [55]. These assays are designed for use in asymptomatic adults aged ≥ 50 years, who represent a population with a rising baseline cancer risk, rather than as a substitute for organ-specific screening. However, they remain investigational and are not yet approved for population-wide screening.

### 6.2. Low-Dose CT Screening and Radiomics

Low-dose computed tomography (LDCT) has emerged as a cornerstone in the early detection of lung cancer, particularly in high-risk populations such as long-term smokers. The National Lung Screening Trial (NLST) in the United States demonstrated a 20% reduction in lung cancer-specific mortality through annual LDCT screening compared to chest radiography [56]. Similarly, the Nederlands-Leuvens Longkanker Screenings Onderzoek (NELSON) trial, conducted in the Netherlands and Belgium, reported a 24% reduction in mortality over a decade of follow-up, with a hazard ratio of approximately 0.76 [49]. These findings form the cornerstone of current lung cancer screening guidelines advocated by the United States Preventive Services Task Force (USPSTF) and the American Cancer Society. Importantly, LDCT screening programs have significantly increased the proportion of lung cancers detected at early stages (I–II), which are more amenable to curative interventions [57]. Consequently, LDCT-based screening initiatives are being scaled globally.

Advances in computational imaging and artificial intelligence (AI) have further augmented the diagnostic yield of LDCT. Radiomics, which involves the high-dimensional quantitative analysis of CT features, such as nodule shape, edge sharpness, and texture, has shown promise in distinguishing benign from malignant pulmonary nodules. Recent studies demonstrate that radiomics-based algorithms not only enhance classification accuracy but can also infer underlying molecular characteristics, including *EGFR* and *KRAS* mutation status, directly from imaging data [58,59,60]. Furthermore, the integration of deep learning models has markedly improved diagnostic precision. In the 4-IN-THE-LUNG-RUN trial, the feasibility of AI as a first-reader (i.e., an automated filter to rule out negative scans before human review) in LDCT lung cancer screening was prospectively evaluated. Among 443 false-negative (FN) scans, AI missed 33 cases while radiologists missed 410, with no overlap. Of the AI FN cases, 9.1% were positive and 90.9% indeterminate; after follow-up, 93.3% were reclassified as negative, and most errors were due to detection (69.7%) or segmentation (30.3%) failures. In contrast, among radiologist FN cases, 90% were later reclassified as negative, and only 2.2% remained positive [61,62].

In addition to AI-assisted LDCT interpretation, machine learning and deep learning approaches have demonstrated substantial utility across multiple facets of NSCLC management. Erdogdu et al. developed ML models incorporating thoracic CT imaging and clinicopathological data to predict N2 lymph node metastasis, achieving up to 95.7% accuracy and outperforming conventional statistical models, highlighting AI’s potential in mediastinal staging and surgical decision-making [63]. Complementarily, Ge et al. demonstrated that deep-learning-based image analysis can accurately and reproducibly quantify PD-L1 expression in IHC-stained NSCLC specimens, reducing inter-observer variability and enabling standardized patient stratification for immunotherapy [64].

A broader systematic review by Bonci et al. confirmed that AI, artificial neural networks, and ML enhance diagnostic precision, prognostic prediction, and treatment personalization, although evidence on patient-reported outcomes and cost-effectiveness remains limited [65]. These studies underscore the expanding applications of AI beyond image-based nodule detection, encompassing staging, molecular phenotyping, and therapy selection, reflecting a transformative role in NSCLC diagnostics and personalized care pathways. Newer AI-enhanced decision support tools, often embedded within computer-aided detection (CAD) systems, are being increasingly deployed to flag suspicious lesions, minimize diagnostic variability, and reduce radiologist burden. As such, radiomics and machine learning represent rapidly advancing fields, with several FDA-cleared algorithms already in clinical use and additional validation efforts underway in ongoing large-scale trials [59].

### 6.3. Novel and Investigational Diagnostic Strategies

While liquid biopsy and imaging remain central pillars of early detection in NSCLC, several emerging diagnostic modalities are expanding the frontiers of non-invasive, highly sensitive cancer detection. These investigational approaches leverage diverse biological sources—ranging from breath and sputum to immune signatures and digital biomarkers—and promise to complement existing methods in the pursuit of earlier and more precise diagnosis. For instance, sputum—an easily accessible sample from the respiratory tract—harbors exfoliated cells and tumor-derived DNA from airway epithelium. Advances in methylation-specific PCR and NGS have enabled the detection of promoter hypermethylation in tumor suppressor genes such as *SHOX2*, *RASSF1A*, *p16 (CDKN2A)*, and *MGMT*, with such epigenetic changes often preceding radiographic evidence [66].

Notably, methylation of CDKN2A was detected in 100% of sputum samples from individuals who later developed squamous cell carcinoma, up to three years before diagnosis [67]. This suggests strong potential for early screening in high-risk populations. Integration with automated cytology and AI-based classification continues to enhance the utility of sputum-based assays at the point of care.

In parallel, the analysis of exhaled breath, commonly referred to as “breathomics,” has gained attention due to its content of volatile organic compounds (VOCs) that reflect metabolic and oxidative changes characteristic of malignancy. In NSCLC, dysregulated cellular metabolism produces unique VOC profiles, which can be detected using platforms like gas chromatography–mass spectrometry (GC-MS) or electronic noses (eNose). Preliminary studies have demonstrated significant diagnostic accuracies in differentiating lung cancer from benign or healthy states [68], highlighting the promise of this rapid and non-invasive tool for population-level screening. However, standardization and external validation remain essential.

Immunogenomic profiling is another promising frontier. The development of cancer elicits measurable changes in host immune surveillance, often before lesions become radiographically apparent. High-throughput sequencing of the peripheral T-cell receptor (TCR) repertoire has revealed clonal expansions associated with the recognition of tumor antigens. Specific TCR signatures are being investigated as early markers of malignancy [69], and combining these with circulating cytokine profiles and immune checkpoint expression may improve both sensitivity and tumor specificity.

Salivary diagnostics also offer a non-invasive alternative for early detection. Saliva contains tumor-derived nucleic acids and proteins for proteomic analyses. Proteomic profiling of saliva using 2D-DIGE and mass spectrometry identified 16 candidate biomarkers for lung cancer, with three showing strong diagnostic potential. These salivary proteins achieved a sensitivity of up to 88.5% and a specificity of 92.3%, highlighting the promise of saliva as a non-invasive diagnostic fluid for early lung cancer detection [70,71]. These tests, if validated, could be particularly well-suited for widespread community screening due to their ease of collection and patient compliance.

**Figure 1:**
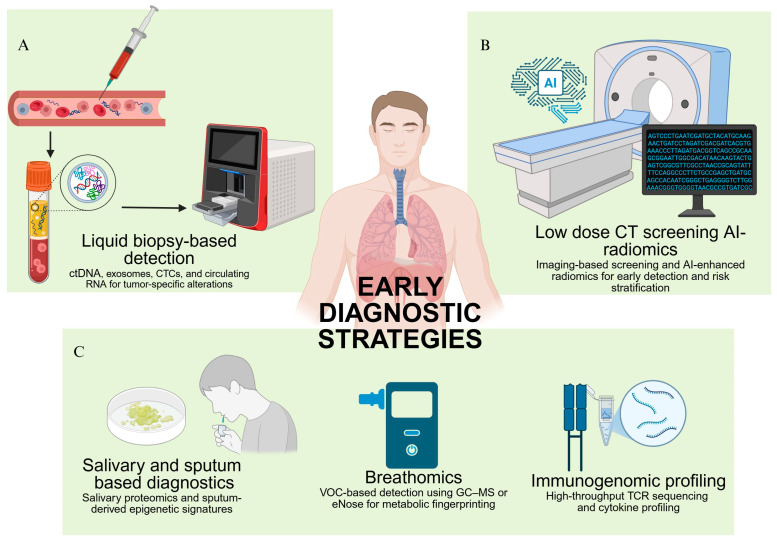
**Multi-modal strategies for early NSCLC diagnosis:** (**A**) *Liquid biopsy-based detection* leverages circulating tumor DNA (ctDNA), exosomes, circulating tumor cells (CTCs), and circulating RNA in blood to detect tumor-specific genomic and epigenomic alterations non-invasively. (**B**) *Low-dose computed tomography (LDCT) with AI-driven radiomics* uses imaging-based screening integrated with artificial intelligence to extract high-dimensional imaging features, improving early detection, risk stratification, and diagnostic accuracy. (**C**) Investigational, minimally invasive approaches include: *Salivary and sputum-based diagnostics*, utilizing salivary proteomics and sputum-derived epigenetic markers to identify early oncogenic changes; *Breathomics*, which involves profiling of volatile organic compounds (VOCs) in exhaled breath via gas chromatography–mass spectrometry (GC–MS) or electronic nose (eNose) technologies for metabolic fingerprinting; and *Immunogenomic profiling*, incorporating high-throughput T-cell receptor (TCR) sequencing and cytokine profiling to detect immune perturbations indicative of early tumor presence.

Finally, a novel dimension of early diagnosis is emerging from the integration of digital health technologies. Digital phenotyping—the continuous collection of behavioral and physiological data from smartphones or wearable devices—is being explored as a passive, real-time strategy for detecting preclinical disease states. Alterations in respiratory patterns, physical activity, and sleep architecture may subtly reflect early systemic shifts induced by tumor growth. Machine learning algorithms are being trained to identify these changes and flag potential concerns, offering a paradigm shift toward continuous, ambient health monitoring [72]. These investigational strategies underscore a move toward multi-modal early detection frameworks that transcend conventional biomarkers. By integrating molecular, metabolic, immune, and digital signals, they hold the potential to not only enhance sensitivity and specificity but also to democratize access to early NSCLC diagnostics across clinical and community settings. Figure 1 represents current multi-modal strategies for early NSCLC diagnosis.

## 7. New Therapeutic Paradigms and Emerging Areas in Treating NSCLC

The therapeutic landscape of NSCLC has undergone a profound transformation in recent years, marked by the advent of precision oncology and immunotherapy. Innovations targeting specific genetic alterations, modulation of the tumor microenvironment, and the emergence of biologics have expanded the arsenal beyond traditional chemotherapy. Several novel agents and combinatorial strategies are now redefining treatment paradigms across disease stages. This section presents a comprehensive synthesis of key advances over the past five years, emphasizing translational relevance and clinical impact. The discussion is organized into thematic subdomains reflecting the diversity and depth of modern NSCLC therapeutics. Table 1 provides a list of novel therapies in NSCLC.

### 7.1. Next-Generation Targeted Therapies

The era of precision oncology in NSCLC has expanded well beyond EGFR and ALK to encompass a broad spectrum of additional oncogenic drivers and targetable mutations. Among the most significant developments is the clinical translation of direct *KRAS* G12C inhibitors. Agents such as sotorasib (AMG 510) and adagrasib (MRTX849) are now FDA-approved for patients with previously treated KRASG12C-mutant NSCLC [73,74]. In the Phase 2 CodeBreaK 100 trial, sotorasib demonstrated an objective response rate (ORR) of approximately 41% and a median progression-free survival of 6.3 months; notably, one-third of treated patients remained alive at 2 years (median overall survival, OS, ~12.5 months) [75]. This data reflects a transformative advance for a mutation class previously considered intractable.

**Table 1:** Novel Therapies in NSCLC.

Modality	Example Agent(s)	Mechanism	Clinical Stage/Approval	Clinical Trials/References
Antibody–Drug Conjugates (ADCs)	Datopotamab deruxtecan (TROP2)	TROP2-targeted cytotoxic delivery	Phase 3	NCT04656652/[76]
Antibody–Drug Conjugates (ADCs)	Trastuzumab deruxtecan (HER2)	HER2-targeted cytotoxic delivery	FDA-approved	NCT03734029/[77]
Antibody–Drug Conjugates (ADCs)	Patritumab deruxtecan (HER3)	HER3-targeted cytotoxic delivery	Phase 2	NCT05865990/[78]
Bispecific Antibodies	Amivantamab (EGFR-MET)	EGFR and MET dual targeting	FDA-approved	NCT02609776/[79]
Bispecific Antibodies	KN046 (PD-L1/CTLA-4)	Dual checkpoint blockade	Phase 2	NCT04054531/[80]
Bispecific Antibodies	SHR-1701 (PD-L1/TGF-β)	Checkpoint + TGF-β blockade	Phase 1	NCT03774979/[81]
CAR-T Cells	EGFR-directed CAR-T	Engineered T cells for EGFR+ tumors	Phase 1	NCT03030001/[82]
CAR-T Cells	MUC1-directed CAR-T	CARs target MUC1-overexpressing NSCLC	Phase 1	NCT03525782/[83]
CAR-T Cells	MSLN-directed CAR-T	CARs target mesothelin+ tumors	Phase 1	NCT02414269/[84]
Oncolytic Virus	MEM-288	IFN-β + CD40L adenoviral vector	Phase 1	NCT05076760/[85]
Oncolytic Virus	DNX-2401 (oncolytic adenovirus)	Oncolysis + immune priming	Phase 2	NCT02798406/[86]
Oncolytic Virus	RP1 (oncolytic HSV)	Oncolysis + GM-CSF expression	Phase 2	NCT03767348/[87]
Cancer Vaccines	BNT116 + Cemiplimab	mRNA vaccine + PD-1 blockade	Phase 1–2	NCT05557591/[88]
Cancer Vaccines	mRNA-4157 + Pembrolizumab	Neoantigen mRNA vaccine + anti-PD-1	Phase 2	NCT06077760/[89]
Cancer Vaccines	TG4010 (MUC1 + IL-2)	Viral vaccine targeting MUC1	Phase 2	NCT00415818/[90]
Immune Checkpoint Blockade	Nivolumab	Anti–PD-1 checkpoint blockade	FDA-approved	NCT02175017/[91]
Immune Checkpoint Blockade	Atezolizumab	Anti–PD-L1 checkpoint blockade	FDA-approved	NCT02409342/[92]
Neoantigen Vaccines	GEN-009 (neoantigen peptides)	Synthetic long peptides + adjuvant	Phase 1	NCT03633110/[93]
Neoantigen Vaccines	NEO-PV-01 + Nivolumab	Tumor-specific peptides + PD-1 inhibitor	Phase 2	NCT03380871/[94]
Neoantigen Vaccines	Personalized mRNA vaccines	mRNA encoding patient-specific neoantigens	Phase 1	NCT03908671/[95]

Beyond G12C, efforts are underway to target other *KRAS* mutations. MRTX1133, a noncovalent inhibitor targeting *KRASG12D*, has demonstrated substantial tumor regression in preclinical NSCLC models [96]. Complementary strategies, including SOS1 inhibitors and pan-*KRAS* inhibitors, are actively being developed to enhance efficacy and overcome tumour resistance. Notably, SOS1 inhibition using agents such as BI-3406 has been shown to improve the efficacy of KRASG12C inhibitors by suppressing adaptive receptor tyrosine kinase/ERK signaling and resensitizing drug-tolerant persister cells, thereby delaying the emergence of resistance, even in the presence of co-mutations like *KEAP1* and *STK11* [97].

*MET* exon 14 skipping mutations—which occur in approximately 3–4% of lung adenocarcinomas—are now effectively treated with MET tyrosine kinase inhibitors such as capmatinib, tepotinib, and savolitinib [98,99,100]. Recent advances in fourth-generation EGFR TKIs are addressing acquired resistance to third-generation inhibitors such as Osimertinib. Mechanisms of resistance, including on-target EGFR-C797S mutations and off-target alterations like MET amplification or HER2 activation, limit long-term efficacy. Novel fourth-generation agents, such as thiazole-amid allosteric inhibitors, have demonstrated high specificity for mutant EGFR and preclinical activity in overcoming resistance. Early phase 1/2 trials are ongoing to evaluate their clinical potential, representing a promising strategy to enhance durable responses in EGFR-mutant NSCLC [101].

As molecularly targeted therapies mature, the emergence of on-therapy resistance has become a major clinical challenge. Understanding the spectrum of acquired resistance mechanisms—ranging from on-target mutations to bypass signaling and histologic transformation—has informed next-generation sequencing (NGS)-guided re-biopsy strategies and adaptive treatment algorithms. Table 2 summarizes contemporary evidence-based approaches to post-progression evaluation and therapeutic decision-making across major oncogenic drivers in NSCLC. This framework highlights how integrating ctDNA profiling, tissue re-biopsy, and combinatorial regimens can help extend the durability of precision oncology.

Clinical trials like GEOMETRY mono-1 and VISION have shown significant efficacy of capmatinib in treatment-naïve patients and tepotinib in pretreated individuals, with additional CNS activity observed across agents [132]. RET fusions, found in 1–2% of NSCLC cases, respond well to selective RET inhibitors, such as selpercatinib and pralsetinib, although resistance can occur via mechanisms like MET amplification. Emerging evidence suggests that combined *RET* and *MET* inhibition—for example, selpercatinib with capmatinib—may overcome *MET*-driven resistance and achieve sustained clinical responses [133]. The landscape has also expanded to include *HER2* mutations, *NTRK* fusions, and less frequent alterations such as *NRG1* and *ErbB3* [134,135]. Bispecific agents and next-generation tyrosine kinase inhibitors (TKIs) are providing robust clinical responses in these genomically defined subsets of lung cancer. The increasing granularity of molecular stratification is enabling rational deployment of precision therapies, thereby benefiting a growing proportion of patients with advanced NSCLC.

### 7.2. Innovations in Immunotherapy and Combinatorial Regimens

Despite remarkable progress with immune checkpoint blockade (ICB), a significant proportion of NSCLC patients either do not respond or ultimately progress, underscoring the need for next-generation immunotherapy strategies. Dual checkpoint inhibition has emerged as one approach, as exemplified by the use of nivolumab plus ipilimumab in PD-L1-positive tumors. Patients with a high tumor mutational burden experienced significantly longer progression-free survival with the combination of these two drugs compared to standard chemotherapy [136]. Furthermore, immune checkpoints such as LAG-3 and TIGIT are being investigated to enhance antitumor immunity. Tiragolumab, a monoclonal antibody targeting TIGIT, has shown encouraging results in clinical trials. In the CITYSCAPE Phase II study for NSCLC, the combination of tiragolumab with a PD-L1 inhibitor improved objective response rates (37% vs. 21%) and progression-free survival compared to PD-L1 blockade alone, highlighting its potential to synergize with existing immunotherapies through complementary mechanisms of T cell activation [137].

However, the subsequent Phase III SKYSCRAPER-01 trial in PD-L1–high, first-line NSCLC did not meet its primary endpoints, as tiragolumab plus atezolizumab failed to show statistically significant improvements in progression-free survival (7.0 vs. 5.6 months; HR 0.78, *p* = 0.02) or overall survival (23.1 vs. 16.9 months; HR 0.87, *p* = 0.22), though numerical trends suggested potential antitumor activity [138]. For patients with unresectable stage III NSCLC harboring *EGFR* mutations, consolidation therapy with osimertinib following definitive chemoradiation has shown a clinically meaningful progression-free survival benefit. The Phase III LAURA trial evaluated osimertinib versus placebo in this setting. It demonstrated a significant extension of median PFS, underscoring the importance of comprehensive molecular profiling even in stage III disease. Importantly, the safety profile of osimertinib was consistent with prior studies, with most adverse events being low-grade, making it a well-tolerated option for patients who have completed chemoradiation and are at high risk for disease recurrence [139].

A critical innovation is the integration of immunotherapy into earlier lines of treatment. The CheckMate-816 trial established neoadjuvant chemoimmunotherapy as a new standard, wherein it demonstrated that adding nivolumab to neoadjuvant chemotherapy improved pathologic complete response rates compared to chemotherapy alone in resectable NSCLC [140]. The IMpower010 trial showed that adjuvant atezolizumab following chemotherapy improved disease-free survival, with emerging overall survival benefit in stage II–IIIa NSCLC with PD-L1 > 50% [141]. These results collectively redefine the treatment paradigm across disease stages. In addition, combinatorial regimens involving chemotherapy, radiation, anti-angiogenic agents, and novel immunomodulators are being investigated to overcome resistance mechanisms.

The ALINA trial evaluated adjuvant alectinib versus platinum-based chemotherapy in patients with completely resected ALK-positive NSCLC (stage IB–IIIA). Alectinib markedly improved disease-free survival compared with chemotherapy and also showed benefit in preventing central nervous system recurrence [142]. Based on these findings, the U.S. Food and Drug Administration approved alectinib (Alecensa) as the first adjuvant treatment for patients with ALK-positive early-stage lung cancer in April 2024. Similarly, The KEYNOTE-671 trial, a global phase 3 study, evaluated the efficacy of neoadjuvant pembrolizumab combined with chemotherapy, followed by adjuvant pembrolizumab, in patients with resectable early-stage NSCLC. The trial demonstrated a significant improvement in overall survival, with a 36-month survival estimate of 71% in the pembrolizumab group compared to 64% in the placebo group. This survival benefit was accompanied by a median event-free survival of 47.2 months in the pembrolizumab group versus 18.3 months in the placebo group. These findings underscore the potential of perioperative pembrolizumab as a new standard of care for patients with resectable early-stage NSCLC [143].

Preclinical models support the addition of epigenetic modifiers, metabolic agents, and even microbiome interventions to enhance the efficacy of ICB. Animal studies have shown that blockade of PD-1 in combination with TIGIT or LAG-3 inhibitors can synergistically improve tumor control. Although many of these approaches remain investigational, they represent a frontier of personalized immunotherapy in NSCLC.

### 7.3. Antibody–Drug Conjugates and Bispecific Antibodies

Antibody-based therapeutics are revolutionizing the management of NSCLC by combining tumor selectivity with potent cytotoxic effects. Among these, antibody–drug conjugates (ADCs) have shown particular promise. The TROPION-Lung01 phase 3 trial established datopotamab deruxtecan (Dato-DXd), a TROP2-targeted ADC, as superior to docetaxel in previously treated NSCLC. Dato-DXd improved progression-free survival (median 4.4 vs. 3.7 months; hazard ratio 0.75; *p* < 0.01), particularly in nonsquamous subtypes, with a manageable safety profile that included ILD in 9% of patients at a severity grade ≥ 3 [144]. These findings suggest that Dato-DXd may offer a clinically meaningful option for patients with advanced nonsquamous NSCLC. However, regulatory decisions are pending, and further studies are needed to confirm these results and establish the role of Dato-DXd in this setting.

Another landmark development is trastuzumab deruxtecan (T-DXd), approved for HER2-mutant NSCLC. The DESTINY-Lung01 phase 2 study reported ORRs of 26–34% in heavily pretreated HER2-positive tumors, establishing T-DXd as the first available therapy for HER2-mutant NSCLC [121]. Several other ADCs targeting HER3, CEACAM5, and *EGFR* are currently in clinical trials.

Bispecific antibodies (bsAbs) represent a novel class of immunotherapeutics designed to simultaneously target two distinct antigens or pathways, offering a multi-pronged approach in the treatment of NSCLC. Amivantamab, an *EGFR-MET* bispecific monoclonal antibody, was FDA-approved in May 2021 for the treatment of adults with locally advanced or metastatic NSCLC harboring *EGFR* exon 20 insertion mutations and is showing promising results [145].

The MARIPOSA trial evaluated first-line amivantamab plus lazertinib versus osimertinib in patients with *EGFR*-mutant advanced NSCLC. The combination therapy prolonged progression-free survival compared with osimertinib and demonstrated benefit across high-risk subgroups, including patients with TP53 co-mutations, detectable baseline circulating tumor DNA, baseline liver metastases, and those without ctDNA clearance on treatment. These findings suggest that dual *EGFR*/*MET* blockade may provide a new standard of care for patients with high-risk *EGFR*-mutant NSCLC [146].

Additional bsAbs, including KN046 (PD-L1/CTLA-4), AK112 (PD-1/VEGF), and SHR-1701 (PD-L1/TGF-β), are progressing through phase 3 development for various NSCLC subtypes [80,147,148]. These bsAbs offer the potential to block redundant pathways and enhance immune activation simultaneously. ADCs and bsAbs are not only expanding therapeutic options but also redefining treatment sequencing in NSCLC. Their ability to deliver high-potency drugs or modulate immune checkpoints in a tumor-selective manner positions them at the forefront of personalized cancer therapy. Table 1 summarizes the recent advances in therapeutic strategies of NSCLC.

### 7.4. Cellular Therapies, Oncolytic Viruses, and Cancer Vaccines

Cell-based and biologic therapies are also beginning to reshape the NSCLC therapeutic landscape. While CAR-T cell (chimeric antigen receptor T-cell) therapy has revolutionized the treatment of hematologic malignancies, its application in lung cancer remains relatively nascent. Early-phase trials targeting *EGFR*, *MSLN* (mesothelin), *PSCA* (prostate stem cell antigen), and *MUC1* have shown preliminary evidence of tumor control, albeit with challenges related to trafficking and the immunosuppressive tumor microenvironment [149,150].

To enhance the therapeutic efficacy and overcome the limitations associated with conventional CAR-T cell therapies, several innovative strategies are under active investigation. These include armored CARs, which are engineered to secrete pro-inflammatory cytokines or express co-stimulatory ligands to bolster anti-tumor activity within the immunosuppressive tumor microenvironment [150,151]; dual CAR-T cells, which incorporate two distinct antigen-recognition domains to improve specificity and reduce antigen escape [152]; and CAR-natural killer (CAR-NK) cells, which offer advantages such as reduced risk of graft-versus-host disease and the ability to mount innate immune responses alongside CAR-mediated cytotoxicity [153].

Vaccine-based therapies for NSCLC are also gaining renewed attention. Oncolytic virotherapy is emerging as a promising therapeutic avenue in NSCLC, particularly for patient’s refractory to immune checkpoint inhibitors (ICIs). MEM-288, a conditionally replicative adenovirus engineered to express human IFN-β and a membrane-stable form of CD40L (MEM40), is currently under investigation in a Phase 1 trial (NCT05076760). Interim results from this first-in-human study show that MEM-288 is well-tolerated, with no dose-limiting toxicities observed. Among response-evaluable patients with advanced NSCLC, 40% demonstrated tumor shrinkage at the injection site, accompanied by increased intratumoral CD8+ T cell infiltration, enhanced T cell clonotype diversity, and systemic immune activation. These early findings support MEM-288’s potential to reshape the immune microenvironment and re-sensitize tumors to subsequent therapies [154]. A combination trial with standard-of-care docetaxel is currently underway [155]. Additional platforms, including oncolytic herpesvirus, vaccinia, and Maraba viruses, are also under investigation as adjuncts to immunotherapy.

The field of cancer vaccines is being reinvigorated. NSCLC’s high mutational burden makes it a suitable candidate for neoantigen-targeted vaccines. Personalized mRNA and peptide vaccines represent a novel immunotherapeutic strategy currently under clinical evaluation, often in combination with checkpoint inhibitors to enhance antitumor responses. One such investigational approach involves BNT116, a personalized mRNA-based cancer vaccine, being evaluated in combination with the PD-1 inhibitor cemiplimab, in patients with advanced NSCLC exhibiting high PD-L1 expression. The ongoing EMPOWERVAX Lung 1 trial is designed to assess the safety, tolerability, and preliminary efficacy of this combination compared to cemiplimab monotherapy (NCT05557591).

**Figure 2:**
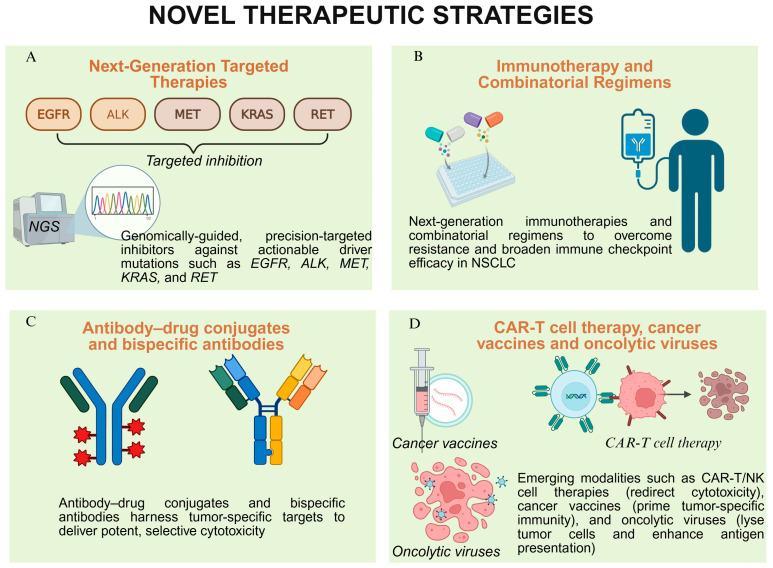
**Novel therapeutic strategies in NSCLC:** (**A**) *Next-generation targeted therapies* utilize genomically guided inhibitors against actionable driver mutations, including *EGFR*, *ALK*, *MET*, *KRAS*, and *RET*, enabling precision oncology based on tumor molecular profiling. (**B**) *Immunotherapy and combinatorial regimens* involve advanced immune checkpoint inhibitors and strategic combinations designed to overcome therapeutic resistance and broaden immunogenicity in NSCLC. (**C**) *Antibody–drug conjugates (ADCs)* and *bispecific antibodies* are engineered to deliver selective cytotoxicity by targeting tumor-specific antigens or simultaneously engaging immune effector cells and tumor cells. (**D**) ***Emerging immunotherapies***, including CAR-T/NK cell therapies (redirecting immune cytotoxicity), cancer vaccines (inducing antigen-specific responses), and oncolytic viruses (lyzing tumor cells and enhancing antigen presentation), represent novel approaches to reprogram the tumor–immune landscape.

Though no vaccine has yet achieved phase 3 success, this strategy remains promising. Contemporary platforms employing mRNA, viral vectors, and synthetic peptides are being tailored to NSCLC. The theoretical appeal lies in “educating” the immune system against tumor-specific neoantigens. Precision targeting of novel oncogenes, enhanced immunotherapy regimens, sophisticated antibody-based therapies, and emerging cell and biologic treatments are collectively redefining the standards of care. These advances are not merely incremental; they constitute a paradigm shift with the potential to significantly extend survival and improve quality of life for patients with NSCLC. Figure 2 represents the current paradigm and evolving landscape of therapeutic strategies in NSCLC.

## 8. Challenges and Future Directions

Despite remarkable advances, the treatment of NSCLC remains fraught with complex challenges rooted in tumor heterogeneity, therapeutic resistance, and the dynamic nature of tumor evolution. Intra-tumoral and inter-patient heterogeneity significantly influence therapeutic responses, as molecular subclones with varying resistance profiles often co-exist within a single tumor. Under selective pressure from targeted therapies or immune checkpoint inhibitors, resistant clones can rapidly expand, leading to relapse even after an initial clinical response. This clonal evolution is driven not only by genetic mutations. But also, by epigenetic alterations, necessitating more flexible and adaptive treatment paradigms.

A major barrier to sustained treatment efficacy is the emergence of drug resistance, both primary and acquired. These resistance mechanisms include secondary mutations in oncogenes (such as *EGFR* T790M and C797S), activation of bypass signaling pathways (e.g., MET amplification), phenotypic transitions, such as the epithelial-to-mesenchymal shift, and the development of an immunosuppressive tumor microenvironment [156]. Addressing this requires serial molecular monitoring and innovative therapeutic strategies—such as drug sequencing, intermittent dosing, or combination approaches—to delay or circumvent resistance.

While current diagnostics predominantly target specific gene alterations, a more comprehensive approach incorporating pan-genomic and epigenomic profiling holds promise for capturing the full landscape of oncogenic drivers and vulnerabilities. Epigenetic markers, including DNA methylation, histone modifications, and non-coding RNAs are increasingly recognized as key determinants of tumor behavior and drug responsiveness. The integration of such data with traditional genomic profiles could enhance patient stratification and uncover novel targets.

Concurrently, advanced multi-omic technologies, including proteomics, single-cell RNA sequencing, and spatial transcriptomics, are revolutionizing our understanding of tumor ecosystems. These tools provide granular insights into cell-to-cell variability, functional protein expression, and the spatial architecture of tumor–immune interactions. When applied longitudinally, they can map tumor evolution in response to therapy, identifying transient or emergent vulnerabilities that may be exploited therapeutically.

Harnessing the full potential of these data-intensive platforms requires robust computational support. AI and machine learning are increasingly being applied to integrate and interpret complex datasets—spanning genomics, imaging, and clinical parameters—to predict treatment outcomes and guide personalized interventions. Moreover, real-world evidence derived from electronic health records and cancer registries is gaining traction as a complementary source of information, enriching clinical trial data and helping to assess therapeutic effectiveness in broader, more diverse patient populations.

Finally, the concept of cancer prevention through immunization is gaining momentum. Drawing inspiration from infectious disease models, researchers are exploring prophylactic and therapeutic cancer vaccines, particularly for individuals at high risk due to genetic susceptibility or chronic carcinogen exposure. Early-phase trials evaluating neoantigen vaccines in the adjuvant setting aim to preempt recurrence by priming the immune system against residual disease. Technological advances, including mRNA vaccine platforms, are accelerating this frontier, offering the potential for a paradigm shift in the prevention and management of NSCLC.

## 9. Conclusions

The therapeutic landscape of NSCLC has expanded dramatically, encompassing targeted agents, immunotherapies, antibody–drug conjugates, and cell-based biologics. These advances, many of which have emerged in just the last five years, are redefining patient outcomes and ushering in an era of individualized oncology. Yet, challenges such as tumor heterogeneity, resistance mechanisms, and the need for better biomarkers persist. Future success will hinge on the integration of multi-omic technologies, AI-enhanced analytics, and real-world clinical data. Moreover, ensuring equitable access to these cutting-edge diagnostics and therapies remains imperative. A multidisciplinary, systems biology approach—bridging molecular pathology, medical oncology, bioinformatics, and public health—will be essential to translate these breakthroughs into durable benefits for all patients with NSCLC.

## Figures and Tables

**Table 2:** Evolving framework for managing on-therapy resistance across key oncogenic subsets in NSCLC *.

On-Therapy Context	Common Resistance Mechanism(s)	What to Test at Progression (Preferred)	Re-Biopsy vs. Liquid Biopsy Strategy	Evidence-Based Next Step(s)	Trial-Ready Options/Notes	References
EGFR classic (Ex19del/L858R) on 1L osimertinib	C797S, L718Q/V, G724S; MET/HER2 amp; KRAS/PIK3CA/NRAS; RET/ALK fusions; SCLC/NE transformation, EMT	Comprehensive NGS (DNA+RNA); MET IHC/ISH; NE markers if histologic shift; ctDNA if tissue hard to access	Start with ctDNA; if negative or histologic change suspected → tissue biopsy; brain imaging for CNS sanctuary	MET amp → add/switch MET TKI; C797S (trans) → 1G/2G EGFR TKI + osimertinib; SCLC transform → platinum–etoposide ± IO; oligoprogression → local therapy + osimertinib	EGFR/MET combos (amivantamab-based), HER3-ADC, 4th-gen EGFR TKIs, EGFR+MET+anti-VEGF triplets	[102,103,104,105]
EGFR exon20 insertion	On-target heterogeneity; MET/HER2 amp; KRAS/PIK3CA; RET fusions	DNA+RNA NGS; ctDNA for rapid spectrum; IHC for HER2	Liquid first; tissue if negative or to quantify amplifications	Amivantamab or mobocertinib; chemo-IO for progression; MET/HER2 bypass → targeted combo	Next-gen exon20 TKIs; EGFR+MET/SHP2/MEK combo trials	[106]
ALK fusion (on 1L alectinib/brigatinib)	G1202R, I1171X, L1196M, compound mutations; MET, KRAS, BRAF bypass; CNS progression	DNA+RNA NGS; ctDNA for mutation spectrum; brain MRI	Liquid detects G1202R/compound variants; tissue if liquid negative	Lorlatinib for G1202R/compound; CNS RT + continue ALK TKI; MET amp → add/switch MET TKI	Next-gen ALK TKIs for compound mutations; SHP2/MEK combos for bypass	[107,108,109]
ROS1 fusion (crizotinib/entrectinib)	G2032R, D2033N, L2026M; CNS progression	DNA+RNA NGS; ctDNA for mutation detection	Liquid first; tissue if liquid negative or discordant	Repotrectinib or lorlatinib for solvent-front mutations; CNS RT for isolated brain progression	Next-gen ROS1 TKIs; pathway combos if bypass identified	[110,111,112]
RET fusion (selpercatinib/pralsetinib)	G810X (solvent-front), gatekeeper; MET, KRAS bypass	DNA+RNA NGS; ctDNA for solvent-front mutations	Liquid first; tissue if negative/discordant	Switch within class if non-solvent-front; MET amp → add/switch MET TKI; chemo-IO as per profile	Next-gen RET TKIs; SHP2/MEK combos for bypass	[112,113,114]
MET exon14 skipping (capmatinib/tepotinib)	D1228, Y1230; KRAS, EGFR, PIK3CA; amplification dynamics	DNA NGS (copy-number), RNA for splice variants; ctDNA for on-target calls	Liquid efficient; tissue for copy-number/alternate drivers	Switch within class if mutation-dependent; combine with EGFR/MEK/PI3K inhibitor per co-alteration	Selective MET TKIs for specific mutants; MET+SHP2/MEK/EGFR combos	[98,115,116]
KRAS G12C (sotorasib/adagrasib)	Y96D/C, H95X, R68S; MET, NRAS, BRAF, FGFR2, PIK3CA; EGFR feedback activation	DNA NGS (on-target & bypass); ctDNA for dynamic resistance tracking	Liquid for speed; tissue if negative or for RNA fusions	Alternate G12C inhibitor for on-target changes; add EGFR blockade; chemo-IO per PD-L1; local therapy for oligoprogression	G12C + SHP2/SOS1/EGFR/MEK combos (CodeBreaK/KRYSTAL); KRAS degraders emerging	[117,118]
BRAF V600E (dabrafenib + trametinib)	MAPK reactivation (NRAS/KRAS), MEK alterations, RTK upregulation	DNA NGS; ctDNA for rapid readout	Liquid acceptable; tissue for RTK amp confirmation	Add/switch MEK/ERK pathway strategies; chemo-IO if no targeted path	ERK inhibitors, RTK blockade triplets under study	[119,120]
HER2 (ERBB2) mutations	Target downregulation, HER2 amp, ADC resistance (payload efflux), PIK3CA	DNA NGS; IHC for HER2 expression; ctDNA to capture heterogeneity	Liquid for mosaicism; tissue to confirm expression/amplification	T-DXd (ADC); switch ADC class; add pathway inhibitor as per co-drivers	HER2 TKIs, PI3K/AKT combos, alternative ADCs	[121,122]
NTRK1/2/3 fusion (larotrectinib/entrectinib)	G595R/G623R (solvent-front), xDFG; MET, BRAF, KRAS bypass	DNA+RNA NGS; ctDNA for on-target	Liquid first; tissue for rare fusion isoforms	Selitrectinib or repotrectinib for solvent-front mutations	Next-gen TRK inhibitors; pathway combos	[123,124,125]
Immunotherapy (PD-1/PD-L1) resistance	STK11/KEAP1, JAK1/2, B2M loss, suppressive TME; EGFR/ALK oncogene-driven immune exclusion	DNA NGS (co-alterations), RNA for inflamed signatures, PD-L1 IHC; ctDNA for TMB dynamics	Tissue preferred for PD-L1 and histology; liquid for mutational context	Switch to targeted therapy if new driver; chemo-IO or IO-IO intensification; trials for STK11/KEAP1	TIGIT/LAG-3/CTLA-4 combos; STING/IL-2/IL-12 agonists; TAM/CAF modulators	[126,127,128,129]
Histologic transformation (EGFR-mutant → SCLC/NE)	RB1/TP53 loss; lineage plasticity	Tissue biopsy with NE IHC (synaptophysin, INSM1), NGS to confirm driver retention	Tissue mandatory (liquid cannot diagnose transformation)	Platinum–etoposide ± IO; continue EGFR TKI for CNS control case-by-case	AURKA, DLL3-ADC, epigenetic therapy trials	[130,131]

* The table outlines predominant resistance mechanisms, preferred molecular testing approaches, optimal use of re-biopsy and liquid biopsy, and evidence-based next-line strategies, with select trial-ready options.

## Data Availability

All data are available within the manuscript.

## Abbreviations

Non-small cell lung cancer, NSCLC; computed tomography, CT; artificial intelligence, AI; transforming growth factor-beta, TGF-β; epidermal growth factor, EGF; fibroblast growth factor, FGF; vascular endothelial growth factor, VEGF; interleukin-1, IL-1; nuclear factor-kB, NF-Kb; vascular endothelial cadherin, VEC; epithelial–mesenchymal transition, EMT; European Respiratory Society, ERS; alveolar epithelial cells, AEC; alpha-smooth muscle actin, α-SMA; endoplasmic reticulum, ER; matrix metalloproteinases, MMP; extracellular signal-regulated kinase, ERK; radiation-induced pulmonary fibrosis, RIPF; T-cell factor/lymphoid enhancer factor, TCF/LEF; Wnt inhibitor 1, WIF1; familial pulmonary fibrosis, FPF; toll interacting protein, TOLLIP; nucleotide polymorphism, SNP; Surfactant Protein C mutation in adult populations, SFTPC.
